# The emerging role of magnesium in CKD

**DOI:** 10.1007/s10157-022-02182-4

**Published:** 2022-01-25

**Authors:** Yusuke Sakaguchi

**Affiliations:** Department of Inter-Organ Communication Research in Kidney Diseases, Osaka University Graduate School of Medicine, 2-2 Yamada-oka, Suita, 565-0871 Japan

**Keywords:** Magnesium, Chronic kidney disease, Vascular calcification, Phosphate, Mortality, Fracture

## Abstract

Increasing evidence has suggested a clinical relevance of magnesium in the context of vascular calcification and mortality among patients with CKD. Hypomagnesemia is not rare among non-dialysis CKD patients despite their decreased glomerular filtration rates; the prevalence rate was about 15% even in CKD stages G4 and G5. Among several potential causes of hypomagnesemia, tubular dysfunction/interstitial fibrosis may play a pivotal role in the development of hypomagnesemia in CKD, which impairs tubular magnesium reabsorption. Magnesium deficiency may, in turn, be involved in the progression of CKD. An in vitro study has revealed that magnesium deficiency aggravates tubular cell death and inflammation induced by phosphate load. In a cohort study of patients with CKD, low-serum magnesium levels enhanced the risk of end-stage kidney disease related to high-serum phosphate levels, suggesting a close relationship between magnesium deficiency and phosphate toxicity. More importantly, magnesium has a potent capacity to inhibit the calcification of vascular smooth muscle cells induced by phosphate. A randomized trial has shown the efficacy of oral magnesium oxide in retarding the progression of coronary artery calcification among non-dialysis CKD patients. Thus, magnesium might provide better cardiovascular prognosis; indeed, hemodialysis patients with mild hypermagnesemia exhibited the lowest mortality rate. Further randomized trials are needed to assess the impact of magnesium in terms of hard clinical outcomes among CKD patients.

## Hypomagnesemia and tubular dysfunction in CKD

Hypomagnesemia is not uncommon among individuals with CKD despite their low glomerular filtration rates. In a cross-sectional study of 5181 patients with CKD stages G1 to G5, hypomagnesemia, defined as serum magnesium levels of less than 1.8 mg/dL, was one of the most prevalent electrolyte abnormalities [1]. Notably, its prevalence did not decline even in CKD stages G4 and G5, where the prevalence rate was approximately 15%. Why do these people suffer from hypomagnesemia?

The mechanisms underlying the development of hypomagnesemia among patients with CKD are diverse. For example, dietary restriction of potassium may limit the intake of magnesium as well, since potassium-rich foods are also rich in magnesium. Urinary magnesium excretion is enhanced by diabetes mellitus, proteinuria, as well as loop and thiazide diuretics. More importantly, tubular dysfunction/interstitial fibrosis might contribute to urinary magnesium loss owing to impaired magnesium reabsorption. Shimizu et al. have reported that in rats with unilateral ureter obstruction, the ureter-ligated kidney showed a dramatic decrease in claudin-16 and transient receptor potential M6, both of which are the major molecules for tubular magnesium reabsorption [2]. In addition, they found an upregulation of claudin-14 which blocks magnesium reabsorption in the thick ascending limb of Henle. As a result, there was a significant decrease in serum magnesium levels accompanied by an increase in fractional excretion of magnesium after releasing the ligation of the ureter.

In line with this animal study, Oka et al. showed a positive correlation between fractional excretion of magnesium and tubular injury markers such as β2-microglobulin among non-dialysis CKD patients [1]. Notably, patients with higher tubular injury markers exhibited a smaller increase in serum magnesium levels after supplementation of magnesium oxide [1], presumably because of an enhanced urinary magnesium excretion due to tubular dysfunction.

## Hypomagnesemia and the risk of CKD progression

Magnesium deficiency, in turn, may be involved in the progression of CKD. In a retrospective cohort study of individuals with diabetic kidney disease, hypomagnesemia was associated with a 2.12-fold higher risk of progression to end-stage kidney disease (95% confidence interval 1.28–3.51; *P* = 0.004) [3]. Although this result may not be surprising given that hypomagnesemia represents a surrogate of tubular injury/interstitial fibrosis, some evidence suggests a direct impact of magnesium on the risk of CKD progression. In heminephrectomized CKD model mice, low-magnesium diet for 6 weeks downregulated α-klotho expression in the kidney and remarkably aggravated tubular injury and interstitial fibrosis induced by high-phosphate diet [4]. In addition, magnesium attenuated phosphate-induced cell death, mitochondrial dysfunction, and inflammation of proximal tubular cells [5]. Consistently, a cohort study of non-diabetic CKD patients has revealed that high serum phosphate levels were associated with an increased risk of progression to end-stage kidney disease only when their serum magnesium levels were low, while the risk was mitigated when their serum magnesium levels were high [5]. Therefore, magnesium might be useful to prevent phosphate-induced kidney injury although this concept should be demonstrated by future interventional studies.

## How does magnesium prevent phosphate-induced kidney injury?

Recently, Shiizaki et al. reported that calcium-phosphate crystals, formed within the proximal tubular lumen, induce tubular damage and interstitial fibrosis by triggering inflammation and cell death [6]. Interestingly, in their animal experiment, urine acidification by ammonium chloride, which inhibits the crystallization of calcium phosphate, attenuated kidney fibrosis. The same mechanism may apply to magnesium because magnesium also possesses a potent inhibitory capacity on calcium phosphate crystallization [7]. Thus, like ammonium chloride, magnesium may prevent kidney injury induced by high phosphate and thereby provide better renal prognosis. To our knowledge, however, no studies have examined the relationship between urinary magnesium concentrations or, more specifically, magnesium concentrations in the proximal tubular lumen and renal prognosis.

## Effects of magnesium on vascular calcification

The inhibitory capacity of magnesium against calcium phosphate crystallization has been well documented in the context of vascular calcification [8]. A lot of in vitro experiments have revealed that magnesium effectively suppresses phosphate-induced calcification of vascular smooth muscle cells [8]. Likewise, in animal studies, high magnesium diet prevents aortic calcification developed in 5/6 nephrectomized mice [9] as well as in Klotho knockout mice [10].

According to these preclinical findings, a randomized controlled trial of 96 patients with non-dialysis CKD stages G3 and G4 assessed the efficacy of oral magnesium oxide on the progression of coronary artery calcification [11]. Over a 2-year study period, serum magnesium levels were significantly elevated from 2.0 to 2.3 mg/dL in the magnesium oxide group, whereas they were not altered in the control group. The percent change in coronary artery calcification score (i.e., Agatston score) was significantly smaller in the magnesium oxide group than in the control group (median; 11.3% vs 39.5%, *P* < 0.001), indicating that magnesium oxide retarded the progression of coronary artery calcification. One of the limitations of this study was the use of magnesium oxide, a well-known laxative, which could modulate the phosphate metabolism via suppressing the intestinal phosphate absorption. This mode of action, which is inherent in many of magnesium compounds, might partly contribute to the inhibition of coronary artery calcification. Tzanakis et al. reported a randomized trial of 72 hemodialysis patients, showing the superiority of magnesium carbonate over calcium carbonate with respect to the progression of arterial calcification [12]. It should be noted, however, that, while coronary artery calcification is associated with an increased risk of cardiovascular events among CKD patients [13], it has yet to be determined whether inhibiting the progression of coronary artery calcification actually improves the prognosis of these patients. Further studies are warranted to evaluate the effect of magnesium on hard cardiovascular endpoints.

## The underlying mechanism of anti-calcification property of magnesium

Although the exact mechanism underlying the anti-calcification property of magnesium has not yet been determined, magnesium is known to suppress the maturation of calciprotein particles (CPPs) which may play a pivotal role in the pathogenesis of vascular calcification.

**Fig. 1:**
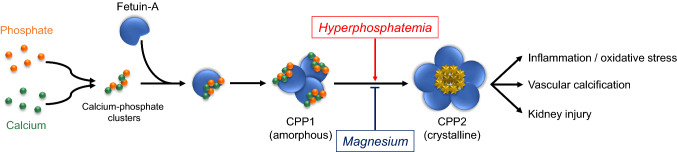
Magnesium inhibits the maturation of calciprotein particles. Calcium and phosphate concentrations in the circulation are supersaturated. Thus, they aggregate to form calcium-phosphate clusters. These clusters bind to fetuin-A to prevent nucleation and crystallization, developing soluble colloidal particle called calciprotein particles (CPPs). CPPs undergo topological changes from amorphous CPP1 to crystalline CPP2 when exposed to a high phosphate environment. Notably, CPP2, but not CPP1, induces calcification of vascular smooth muscle cells as well as inflammation/oxidative stress and may also be involved in kidney injury. Thus, CPP2 is considered as a culprit of phosphate toxicity. Magnesium is a potent inhibitor of CPPs maturation. This is because magnesium ions can substitute for calcium ions in the hydroxyapatite structure. This crystallographic property of magnesium helps prevent the maturation of CPPs and may act as a remedy for phosphate toxicity. *CPPs* calciprotein particles

Calcium and phosphate concentrations in the circulation are supersaturated (Fig. 1). To prevent nucleation and crystallization, they form soluble colloidal particles, CPPs, with mineral-binding proteins like fetuin-A. Since naked calcium-phosphate crystals induce inflammation and oxidative stress when exposed to macrophages [14], covering the mineral crystals with fetuin-A is considered as a biologically protective process. However, CPPs undergo topological changes from amorphous CPP1 to crystalline CPP2 in a high phosphorus environment [15]. Notably, CPP2, but not CPP1, induces calcification of vascular smooth muscle cells as well as inflammation/oxidative stress [16] and may also be a cause of kidney injury [6]. Thus, CPP2 is considered as a culprit of phosphate toxicity. Inhibiting the maturation of CPP1 to CPP2 is theoretically a promising strategy for the prevention of vascular calcification.

Here, magnesium has been shown to suppress the maturation of CPPs. It is well known that magnesium ions can substitute for calcium ions in the hydroxyapatite structure [17]. Since the radius of a magnesium ion (0.069 nm) is smaller than that of a calcium ion (0.099 nm), the substitution of magnesium leads to distortion of the hydroxyapatite lattice and, thereby, loss of crystallinity. This crystallographic property of magnesium helps prevent the maturation of CPPs and may suppress vascular calcification as well as inflammation.

Several randomized trials have reported the efficacy of magnesium for improving serum calcification propensity, T50 [18, 19]. T50 represents the time taken to convert from CPP1 to CPP2 in serum, assessing the integrated ability of serum to resist the maturation of CPPs [15]. T50 is known to be associated with the progression of coronary artery calcification as well as mortality [20–22]. In a double-blind randomized controlled trial of 57 hemodialysis patients, Bressendorff et al. examined the effect of high-magnesium dialysate on T50 [18]. They found a significant increase in T50 among patients dialyzed with high-magnesium dialysate (dialysate magnesium concentration of 2.0 mEq/L) compared to those dialyzed with low-magnesium dialysate (dialysate magnesium concentration of 1.0 mEq/L), indicating that high-magnesium dialysate is useful in attenuating calcification stress. Interestingly, in a post hoc analysis of this trial, the high-magnesium dialysate significantly decreased not only CPPs but also inflammatory cytokines such as tumor necrosis factor-alfa and interleukin-6 [23]. This latter finding may be explained by in vitro evidence showing that CPP2 triggers inflammation [14].

Similarly, the same authors have reported an improvement of T50 after administration of magnesium hydroxide for patients with CKD stages G3 and G4 [19].

Taken together, these randomized trials suggest that magnesium prevents the progression of vascular calcification via antagonizing the maturation of CPPs.

## Serum magnesium levels and mortality in hemodialysis patients

Magnesium might provide better clinical outcomes by preventing the progression of vascular calcification. In a cohort study of 142,555 hemodialysis patients from the Japanese Society for Dialysis Therapy-Renal Data Registry, lower pre-dialysis serum magnesium levels were associated with a higher risk of 1-year mortality [24]. Those with mild hypermagnesemia, i.e., serum magnesium levels of 2.7–3.0 mg/dL, showed the best survival. Unexpectedly, serum magnesium levels of ≥ 3.1 mg/dL were associated with higher mortality although the causality of this association is unknown. There may be some confounders between mild hypermagnesemia and better survival. For example, mild hypermagnesemia may be a surrogate of good nutritional status or medical supplementation of magnesium oxide, which reflect a better overall management of dialysis patients. Well-designed randomized trials are needed to determine the optimal serum magnesium levels for hemodialysis patients.

**Fig. 2:**
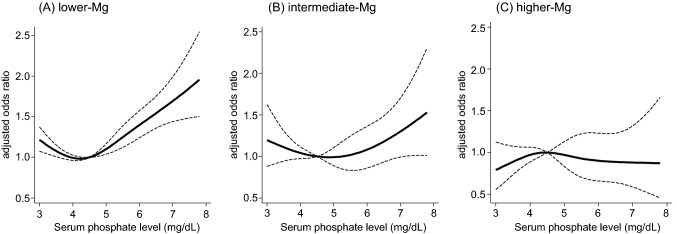
Effect modification by serum magnesium levels on the association between serum phosphate levels and cardiovascular mortality. **A** Among hemodialysis patients with lower serum magnesium levels (< 2.7 mg/dL), higher serum phosphate levels are strongly associated with the risk of cardiovascular mortality. However, this association is attenuated among those with serum magnesium levels of 2.7–3.0 mg/dL (**B**), and disappears among those with serum magnesium levels of 3.1 mg/dL or greater (**C**). There is a significant interaction between magnesium and phosphate on the cardiovascular mortality (*P* for interaction = 0.03). Cited from Ref.25; Sakaguchi Y et al. PLoS ONE 2014; 9(12): e116273. *Mg* magnesium

Given that magnesium counteracts the detrimental effect of phosphate, a close interaction between serum magnesium and phosphate levels can be assumed with respect to the risk of cardiovascular outcomes. In fact, the association between serum phosphate levels and cardiovascular deaths was significantly modified by serum magnesium levels. Among individuals with lower serum magnesium levels, hyperphosphatemia was remarkably associated with an increased risk of cardiovascular mortality, whereas there was no significant association between serum phosphate levels and cardiovascular mortality among those with higher serum magnesium levels (Fig. 2) [25]. This finding further supports the notion that magnesium would benefit especially individuals with hyperphosphatemia.

## Magnesium and the risk of fracture

Although mild hypermagnesemia may be preferable for hemodialysis patients in terms of survival, there are some concerns about its potential harmfulness to the bone metabolism. While magnesium is an essential bone mineral, excess magnesium disturbs bone mineralization. A case series of hemodialysis patients diagnosed with osteomalacia reported that converting high-magnesium dialysate (1.5 mEq/L) to low-magnesium dialysate (0.5 mEq/L) restored bone mineralization [26]. This might imply that a high magnesium state is not suitable for bone mineralization.

According to the data from the Japanese Society for Dialysis Therapy-Renal Data Registry, however, mild hypermagnesemia was not associated with an increased risk of hip fracture of hemodialysis patients [27]. Rather, lower serum magnesium levels were significantly associated with an increased risk of hip fracture. Notably, the population attributable fraction of serum magnesium levels for incident hip fracture was higher than that of serum calcium, serum phosphate, and intact parathyroid hormone levels, suggesting that low magnesium may contribute to the fracture burden more deeply than the other factors related to mineral and bone disorder in CKD. Further studies are needed to ensure whether the intervention for magnesium really prevents the occurrence of fracture.

## Ionized magnesium levels among hemodialysis patients

The physiologically active form of magnesium in the body is ionized magnesium. According to a cross-sectional study of 118 Japanese hemodialysis patients, ionized magnesium levels of hemodialysis patients may not be so elevated compared to total magnesium levels [28]; the prevalence of hypermagnesemia defined by total serum magnesium levels was 69%, whereas that defined by ionized magnesium level was only 13% [28]. The mean ionized magnesium level was 0.54 mM, which was within the reference range (0.45–0.60 mM). The ionized-to-total magnesium ratio was lower in patients on hemodialysis than that in non-dialysis patients (51% vs 63%, *P* < 0.001). Interestingly, the ionized-to-total magnesium ratio of hemodialysis patients was inversely correlated with the anion gap and serum phosphate levels, suggesting that a variety of anions accumulated in the blood may form a complex with magnesium and contribute to the reduction in the ionized fraction of magnesium. As a result, ionized magnesium levels of hemodialysis patients were mostly within the reference range despite their elevated total serum magnesium levels. Among hemodialysis patients, therefore, it seems reasonable to target mild hypermagnesemia in order to maintain ionized magnesium levels within the reference range.

## Conclusions

Compelling evidence supports the clinical relevance of magnesium among patients with CKD especially in terms of vascular calcification and mortality. However, there are still many issues that have not been fully realized. For example: “what is the truly appropriate target range of serum magnesium levels?” and “what is the best approach to increase serum magnesium levels?” There is also an urgent need to develop an effective measure for refractory hypomagnesemia. More randomized trials are required to solve these questions and establish an optimal strategy to manage magnesium for improving the prognosis of patients with CKD.

## References

[1] Oka T, Hamano T, Sakaguchi Y, Yamaguchi S, Kubota K, Senda M (2019). Proteinuria-associated renal magnesium wasting leads to hypomagnesemia: a common electrolyte abnormality in chronic kidney disease. Nephrol Dial Transplant.

[2] Shimizu T, Takayanagi K, Iwashita T, Ikari A, Anzai N, Okazaki S (2018). Down-regulation of magnesium transporting molecule, claudin-16, as a possible cause of hypermagnesiuria with the development of tubulo-interstitial nephropathy. Magnes Res.

[3] Sakaguchi Y, Shoji T, Hayashi T, Suzuki A, Shimizu M, Mitsumoto K (2012). Hypomagnesemia in type 2 diabetic nephropathy: a novel predictor of end-stage renal disease. Diabetes Care.

[4] Sakaguchi Y, Hamano T, Matsui I, Oka T, Yamaguchi S, Kubota K (2019). Low magnesium diet aggravates phosphate-induced kidney injury. Nephrol Dial Transplant.

[5] Sakaguchi Y, Iwatani H, Hamano T, Tomida K, Kawabata H, Kusunoki Y (2015). Magnesium modifies the association between serum phosphate and the risk of progression to end-stage kidney disease in patients with non-diabetic chronic kidney disease. Kidney Int.

[6] Shiizaki K, Tsubouchi A, Miura Y, Seo K, Kuchimaru T, Hayashi H (2021). Calcium phosphate microcrystals in the renal tubular fluid accelerate chronic kidney disease progression. J Clin Invest.

[7] Ter Braake AD, Eelderink C, Zeper LW, Pasch A, Bakker SJL, de Borst MH (2020). Calciprotein particle inhibition explains magnesium-mediated protection against vascular calcification. Nephrol Dial Transplant.

[8] Ter Braake AD, Shanahan CM, de Baaij JHF (2017). Magnesium counteracts vascular calcification: passive interference or active modulation?. Arterioscler Thromb Vasc Biol.

[9] Diaz-Tocados JM, Peralta-Ramirez A, Rodríguez-Ortiz ME, Raya AI, Lopez I, Pineda C, Herencia C (2017). Dietary magnesium supplementation prevents and reverses vascular and soft tissue calcifications in uremic rats. Kidney Int.

[10] Ter Braake AD, Smit AE, Bos C, van Herwaarden AE, Alkema W, van Essen HW (2020). Magnesium prevents vascular calcification in Klotho deficiency. Kidney Int.

[11] Sakaguchi Y, Hamano T, Obi Y, Monden C, Oka T, Yamaguchi S (2019). A randomized trial of magnesium oxide and oral carbon adsorbent for coronary artery calcification in predialysis CKD. J Am Soc Nephrol.

[12] Tzanakis IP, Stamataki EE, Papadaki AN, Giannakis N, Damianakis NE, Oreopoulos DG (2014). Magnesium retards the progress of the arterial calcifications in hemodialysis patients: a pilot study. Int Urol Nephrol.

[13] Chen J, Budoff MJ, Reilly MP, Yang W, Rosas SE, Rahman M (2017). Coronary artery calcification and risk of cardiovascular disease and death among patients with chronic kidney disease. JAMA Cardiol.

[14] Smith ER, Hanssen E, McMahon LP, Holt SG (2013). Fetuin-A-containing calciprotein particles reduce mineral stress in the macrophage. PLoS ONE.

[15] Pasch A, Farese S, Gräber S, Wald J, Richtering W, Floege J (2012). Nanoparticle-based test measures overall propensity for calcification in serum. J Am Soc Nephrol.

[16] Aghagolzadeh P, Bachtler M, Bijarnia R, Jackson C, Smith ER, Odermatt A (2016). Calcification of vascular smooth muscle cells is induced by secondary calciprotein particles and enhanced by tumor necrosis factor-α. Atherosclerosis.

[17] Laurencin D, Almora-Barrios N, de Leeuw NH, Gervais C, Bonhomme C, Mauri F (2011). Magnesium incorporation into hydroxyapatite. Biomaterials.

[18] Bressendorff I, Hansen D, Schou M, Pasch A, Brandi L (2018). The effect of increasing dialysate magnesium on serum calcification propensity in subjects with end stage kidney disease: a randomized, controlled clinical trial. Clin J Am Soc Nephrol.

[19] Bressendorff I, Hansen D, Schou M, Silver B, Pasch A, Bouchelouche P (2016). Oral magnesium supplementation in chronic kidney disease stages 3 and 4: efficacy, safety, and effect on serum calcification propensity-a prospective randomized double-blinded placebo-controlled clinical trial. Kidney Int Rep.

[20] Bundy JD, Cai X, Scialla JJ, Dobre MA, Chen J, Hsu CY (2019). Serum calcification propensity and coronary artery calcification among patients with CKD: The CRIC (Chronic Renal Insufficiency Cohort) Study. Am J Kidney Dis.

[21] Pasch A, Block GA, Bachtler M, Smith ER, Jahnen-Dechent W, Arampatzis S (2017). Blood calcification propensity, cardiovascular events, and survival in patients receiving hemodialysis in the EVOLVE trial. Clin J Am Soc Nephrol.

[22] Eelderink C, Te Velde-Keyzer CA, Frenay AS, Vermeulen EA, Bachtler M, Aghagolzadeh P (2020). Serum calcification propensity and the risk of cardiovascular and all-cause mortality in the general population: the PREVEND study. Arterioscler Thromb Vasc Biol.

[23] Bressendorff I, Hansen D, Pasch A, Holt SG, Schou M, Brandi L (2021). The effect of increasing dialysate magnesium on calciprotein particles, inflammation and bone markers: post hoc analysis from a randomized controlled clinical trial. Nephrol Dial Transplant.

[24] Sakaguchi Y, Fujii N, Shoji T, Hayashi T, Rakugi H, Isaka Y (2014). Hypomagnesemia is a significant predictor of cardiovascular and non-cardiovascular mortality in patients undergoing hemodialysis. Kidney Int.

[25] Sakaguchi Y, Fujii N, Shoji T, Hayashi T, Rakugi H, Iseki K (2014). Magnesium modifies the cardiovascular mortality risk associated with hyperphosphatemia in patients undergoing hemodialysis: a cohort study. PLoS ONE.

[26] Gonella M, Ballanti P, Della Rocca C, Calabrese G, Pratesi G, Vagelli G (1988). Improved bone morphology by normalizing serum magnesium in chronically hemodialyzed patients. Miner Electrolyte Metab.

[27] Sakaguchi Y, Hamano T, Wada A, Hoshino J, Masakane I (2018). Magnesium and risk of hip fracture among patients undergoing hemodialysis. J Am Soc Nephrol.

[28] Sakaguchi Y, Hamano T, Kubota K, Oka T, Yamaguchi S, Matsumoto A (2018). Anion gap as a determinant of ionized fraction of divalent cations in hemodialysis patients. Clin J Am Soc Nephrol.

